# Circulating Tumor DNA in Bladder Cancer: Current Clinical Evidence and Emerging Multi-Omics Perspectives—A Narrative Review

**DOI:** 10.3390/cancers18172816

**Published:** 2026-08-31

**Authors:** Mariam Grazia Polito, Luisana Sisca, Davide Caruso, Antonella Cosimati, Carla Adriana Ramirez Reinaga, Etien Leka, Daniele Santini, Gian Paolo Spinelli

**Affiliations:** 1 UOC Oncologia Territoriale, ASL Latina, CDS Aprilia, La Sapienza Università Di Roma Polo Pontino, 04100 Latina, Italy; 2 Department of Medical Oncology, Fondazione Policlinico Universitario Campus Bio-Medico, 00128 Rome, Italy; 3 Department of Translational Research, Institut Curie, 26 Rue d’Ulm, 75005 Paris, France; 4 Department of Medical and Surgical Science and Biotechnology, Università “La Sapienza”, 00161 Rome, Italy

**Keywords:** muscle-invasive bladder cancer, circulating tumor DNA, liquid biopsy, minimal residual disease, biomarkers, immunotherapy, precision oncology

## Abstract

**Background:** Circulating tumor DNA (ctDNA) analysis has emerged as a promising tool for real-time disease monitoring in muscle-invasive bladder cancer (MIBC). This narrative review summarizes current clinical evidence regarding ctDNA across disease stages. **Methods:** We examine recent translational and clinical findings, incorporating key prospective data from practice-changing trials, as well as insights into minimal residual disease (MRD) detection, treatment escalation and de-escalation strategies, and systemic barriers to adoption. **Results:** Postoperative ctDNA positivity consistently identifies patients with molecular residual disease (MRD) who face a substantially higher risk of recurrence and mortality, frequently preceding radiographic relapse by several months. Prospective evidence now validates ctDNA as a predictive biomarker to guide adjuvant immunotherapy escalation, while sustained ctDNA negativity correlates with high long-term disease-free survival. Beyond plasma ctDNA, emerging multi-compartment liquid biopsies—integrating urinary tumor DNA (utDNA)—demonstrate enhanced sensitivity, particularly in bladder-sparing and local surveillance settings. Furthermore, integrating genomic ctDNA profiling with novel post-transcriptional layers like epitranscriptomics offers a functional framework to capture tumor adaptation under therapeutic pressure. However, clinical translation remains constrained by a lack of assay harmonization, variable analytical sensitivity, and the need for standardized intervention thresholds. **Conclusions:** Longitudinal liquid biopsies are rapidly shifting MIBC management from static, stage-based paradigms toward dynamic, molecularly informed precision oncology. While ctDNA-guided strategies show robust clinical utility, prospective interventional validation and technical standardization are required before widespread routine integration.

## Simple Summary

Muscle-invasive bladder cancer is an aggressive disease in which many patients remain at risk of recurrence despite surgery and standard treatments. There is an urgent need for better tools to identify patients who may benefit from additional therapies or, conversely, avoid unnecessary treatment-related toxicity. Circulating tumor DNA, a small amount of tumor-derived genetic material detectable in blood, has emerged as a promising marker for monitoring residual disease and predicting treatment response. In this review, we summarize the current evidence on the clinical applications of circulating tumor DNA in muscle-invasive bladder cancer, including its role in prognosis, treatment selection, and surveillance. We also discuss future perspectives for integrating molecular monitoring into personalized cancer care.

## 1. Introduction

Bladder cancer (BC) is one of the most common malignancies of the urinary tract and represents a major global health burden, accounting for more than 600,000 new cases and over 220,000 deaths annually worldwide [1]. Despite substantial advances in surgical techniques, systemic therapies, and molecular characterization, disease recurrence and progression remain frequent, representing significant challenges to patient management and underscoring the need for more sensitive and dynamic biomarkers to improve risk stratification and guide therapeutic decision-making. In this context, there is a growing shift toward minimally invasive, longitudinal biomarkers capable of capturing tumor evolution in real time.

Muscle-invasive bladder cancer (MIBC) accounts for approximately 25–30% of newly diagnosed bladder cancer cases and is defined by tumor invasion into or beyond the detrusor muscle, conferring a substantially higher risk of local progression and distant metastasis compared with non-muscle-invasive disease [2].

Standard-of-care management typically combines neoadjuvant cisplatin-based chemotherapy with radical cystectomy and pelvic lymphadenectomy or, in selected patients, trimodal bladder-preserving therapy; nevertheless, up to 50% of patients experience disease recurrence, most frequently within the first two years after treatment [3].

This substantial residual risk, together with the limited capacity of conventional imaging to detect microscopic disease, underlies the rationale for molecular tools such as ctDNA that can refine risk stratification beyond pathological staging alone. Current clinical management of BC relies on cystoscopy, urine cytology, imaging, and histopathological evaluation. However, these approaches have important limitations. Cystoscopy is invasive, uncomfortable for patients, and associated with considerable healthcare costs, whereas urine cytology has limited sensitivity, particularly for low-grade tumors. Furthermore, conventional imaging modalities are often unable to detect minimal residual disease (MRD) or identify disease recurrence at its earliest stages. These limitations further support the increasing interest in the development of minimally invasive biomarkers capable of capturing the molecular landscape of bladder cancer in real time [4].

Among liquid biopsy approaches, circulating tumor DNA (ctDNA), a fraction of cell-free DNA released into the bloodstream by apoptotic, necrotic, or actively secreting tumor cells, has emerged as one of the most promising biomarkers across multiple malignancies. Recent advances in next-generation sequencing (NGS) technologies and highly sensitive molecular assays have enabled the detection of tumor-specific genomic alterations in plasma with increasing sensitivity and specificity, opening new opportunities for disease monitoring and personalized treatment strategies [5]. In bladder cancer, ctDNA analysis has shown potential clinical applications including early detection, MRD assessment, prediction of recurrence, monitoring of treatment response, and early identification of mechanisms of therapeutic resistance [6]. The clinical utility of ctDNA is particularly promising in muscle-invasive bladder cancer (MIBC). Several studies have demonstrated that ctDNA positivity following radical cystectomy or neoadjuvant chemotherapy is strongly associated with an increased risk of relapse and inferior survival outcomes, frequently preceding radiographic evidence of recurrence by several months [7]. Furthermore, new data revealed that ctDNA can identify patients with molecular residual disease who derive significant benefit from adjuvant immune-checkpoint inhibitors, suggesting that ctDNA may also serve as a predictive tool for treatment selection [8].

Similarly, in the metastatic setting, longitudinal ctDNA profiling may provide valuable insights into tumor evolution, clonal dynamics, and treatment efficacy, potentially enabling more timely therapeutic interventions and more personalized management [9]. Nevertheless, several challenges remain before ctDNA can be routinely implemented in clinical practice, including assay standardization, variability in analytical sensitivity, optimal timing of sample collection, integration into existing clinical workflows, and the availability of high-level evidence from randomized clinical trials [6].

In this narrative review, we summarize the current evidence regarding the use of ctDNA in bladder cancer, discussing its biological basis, available detection technologies, and clinical applications across different disease stages. We also address the major limitations and future perspectives, with particular emphasis on the role of ctDNA in MRD detection and its potential to advance precision oncology and improve patient outcomes.

Compared with previous reviews and meta-analyses, which mainly focused on the prognostic significance of ctDNA or summarized early evidence from retrospective and exploratory studies, the present review provides an updated and clinically oriented overview of the evolving role of ctDNA in muscle-invasive bladder cancer. By incorporating the most recent prospective clinical trials, this review highlights the transition of ctDNA from a prognostic biomarker toward a potential tool for treatment personalization, including escalation and de-escalation strategies, adjuvant therapy selection, and surveillance optimization.

**Figure 1:**
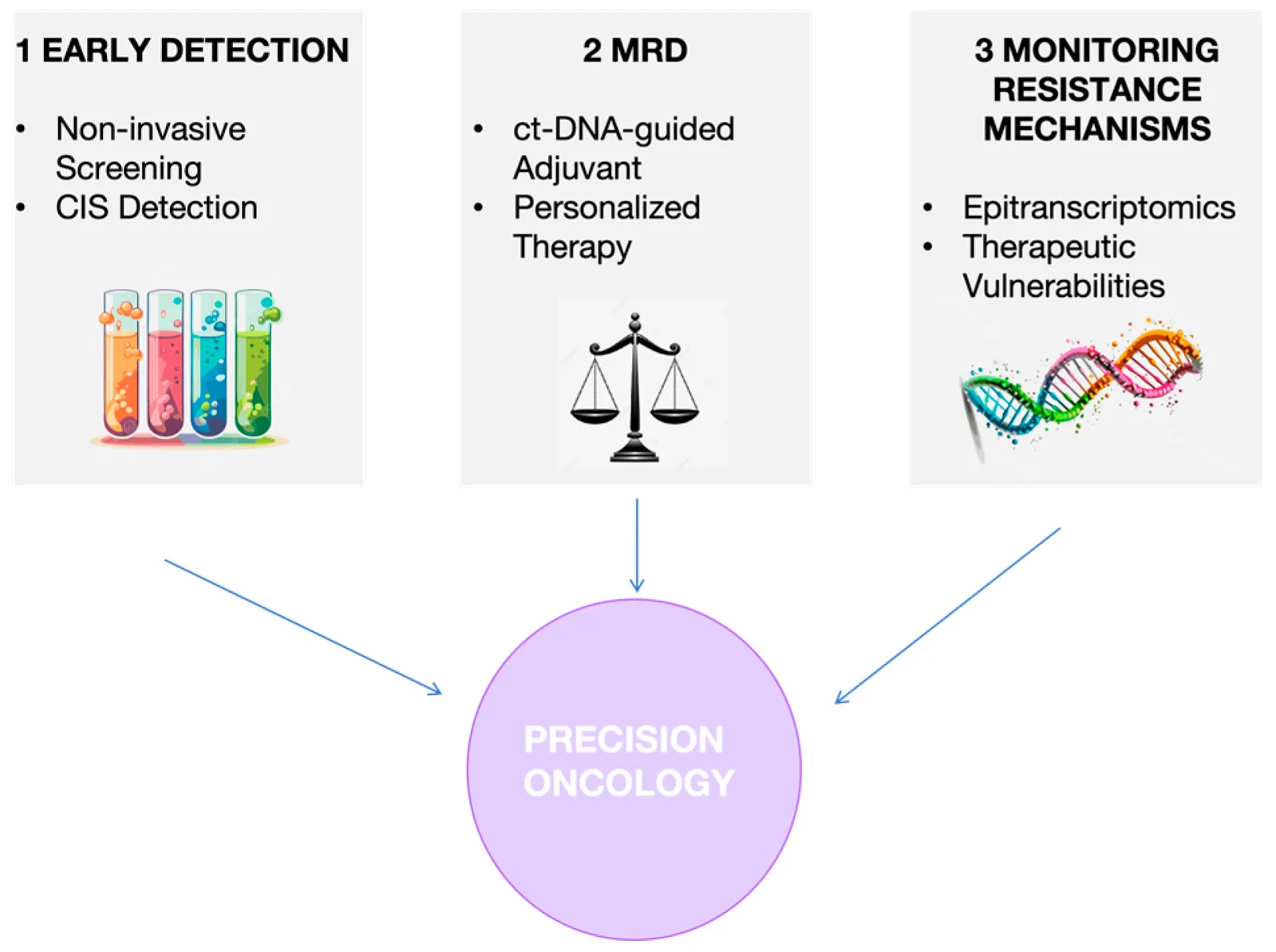
Clinical applications of circulating tumor DNA and multi-omic liquid biopsies driving precision oncology in bladder cancer. Abbreviations: CIS, carcinoma in situ; ctDNA, circulating tumor DNA; MRD, minimal residual disease.

An overview of the main clinical applications of ctDNA across different disease settings in bladder cancer is provided in Figure 1, while Table 1 summarizes the prospective clinical studies that have evaluated ctDNA across the disease continuum.

## 2. ctDNA as a Biomarker of Minimal Residual Disease and Clinical Outcome in Bladder Cancer

Accumulating evidence supports the role of circulating tumor DNA (ctDNA) as both a prognostic and predictive biomarker in muscle-invasive bladder cancer (MIBC). Postoperative ctDNA positivity is consistently associated with a significantly higher risk of recurrence and worse survival outcomes, reflecting the presence of minimal residual disease (MRD). In addition, ctDNA may help identify patients who derive the greatest benefit from adjuvant immunotherapy, whereas those with undetectable ctDNA generally experience more favorable outcomes regardless of treatment exposure. Importantly, ctDNA positivity often precedes radiological recurrence by several months, supporting its role as an early marker of molecular relapse [7,10].

In this context, exploratory long-term ctDNA analyses from the CheckMate 274 trial further suggest that ctDNA status may refine postoperative risk stratification and inform benefit from adjuvant immune checkpoint inhibition. In this analysis, ctDNA was detectable after radical surgery in 40.6% of patients and was strongly associated with shorter disease-free survival (HR 0.30, 95% CI 0.18–0.48). Importantly, ctDNA-positive patients derived greater benefit from adjuvant nivolumab compared with placebo (HR 0.35, 95% CI 0.18–0.66), supporting a potential predictive role for ctDNA in treatment selection, although prospective validation remains required [11].

In line with this concept, the TOMBOLA trial further confirmed the clinical utility of tumor-informed ctDNA monitoring. In this study, 178 evaluable patients were followed longitudinally, and 58% became ctDNA-positive within two years after surgery, with ctDNA detection preceding radiographic recurrence by a median of approximately 90 days [12].

More recently, the phase III IMvigor011 trial provided the first prospective evidence supporting ctDNA-guided adjuvant therapy in muscle-invasive bladder cancer. In this double-blind, randomized study, 761 patients were enrolled and monitored with serial ctDNA testing for up to 1 year after radical surgery; 250 patients who became ctDNA-positive were randomized to receive atezolizumab or placebo. The trial demonstrated significantly improved DFS with atezolizumab (HR 0.64; 95% CI 0.47–0.87; *p* = 0.005) and a median OS of 32.8 versus 21.1 months (HR 0.59; 95% CI 0.39–0.90; *p* = 0.01). Importantly, among patients who remained persistently ctDNA-negative, disease-free survival was 88% at 2 years without treatment, reinforcing the strong favorable prognostic value of sustained ctDNA negativity. Overall, these findings establish ctDNA as a dynamic biomarker capable of guiding post-cystectomy therapeutic decisions based on molecular relapse rather than radiographic disease [13]. Exploratory analyses further indicate that ctDNA kinetics, including early clearance and quantitative burden, may provide additional prognostic refinement beyond binary positivity alone [14].

The prognostic value of ctDNA is further supported in the neoadjuvant setting. In the ABACUS trial, which evaluated atezolizumab in cisplatin-ineligible or -refusing patients with MIBC, ctDNA closely reflected treatment response and long-term clinical outcomes. ctDNA positivity declined from 63% at baseline to 47% after neoadjuvant therapy and to 14% after surgery, highlighting a progressive molecular clearance associated with therapeutic response. Notably, no disease relapses were observed among patients who were ctDNA-negative after neoadjuvant treatment, suggesting a strong association between ctDNA clearance and durable disease control [15].

Importantly, the definition and quantitative interpretation of ctDNA positivity are not fully harmonized across studies. Although a threshold of approximately 4.0 MTM/mL has been used in some quantitative analyses, this value should not be considered a universal biological or clinical cutoff. In several prospective MIBC studies using tumor-informed assays, ctDNA positivity is primarily defined according to the detection of a predefined number of patient-specific tumor variants, while MTM/mL is used as a quantitative measure of circulating tumor burden and its longitudinal change. Therefore, detectable ctDNA values below 4.0 MTM/mL should not automatically be interpreted as clinically irrelevant or equivalent to ctDNA negativity. Low-level detectable ctDNA may represent molecular residual disease, particularly when confirmed longitudinally or when accompanied by an increasing trend. Thus, serial measurements and the direction of ctDNA change may be more informative than application of a single universal quantitative threshold [7,8,11,12,13,14,16]. Table 2 summarizes the principal ctDNA definitions and quantitative approaches used across the prospective studies.

These findings are corroborated by multiple meta-analyses in urothelial carcinoma populations treated with immune checkpoint inhibitors. In a pooled analysis of nine studies, baseline ctDNA positivity was associated with significantly shorter DFS [17]. A larger meta-analysis confirmed these associations, showing that elevated baseline ctDNA levels consistently predict worse DFS and OS across different clinical contexts, detection platforms, and sampling time points. Patients who showed a decrease or clearance in ctDNA levels also had more favorable outcomes [18].

A recent systematic review and meta-analysis further consolidate the robust prognostic value of circulating tumor DNA (ctDNA) in muscle-invasive bladder cancer treated with radical cystectomy. Across more than 1400 patients, ctDNA positivity was associated with substantially higher risks of both recurrence and mortality compared with ctDNA-negative status. Importantly, while adjuvant immunotherapy appeared to confer greater survival benefit in ctDNA-positive patients, its impact was limited in ctDNA-negative disease [19].

Finally, translational evidence from metastatic urothelial carcinoma provides additional insights into the potential role of circulating tumor DNA (ctDNA) as a biomarker of treatment response, although its clinical utility in this setting remains not fully established. In a retrospective exploratory analysis of the phase III KEYNOTE-361 trial, baseline ctDNA was significantly associated with best overall response, PFS, and OS in patients treated with pembrolizumab (*p* = 0.009, *p* < 0.001, and *p* < 0.001, respectively), whereas no significant associations were observed in patients receiving chemotherapy. In addition, early on-treatment changes in ctDNA showed stronger associations with response and survival outcomes under pembrolizumab compared with chemotherapy, despite chemotherapy inducing larger absolute ctDNA reductions from baseline to early treatment cycles. However, when modeled alongside radiographic response, ctDNA dynamics did not demonstrate independent prognostic value for OS, suggesting that their incremental contribution may be context-dependent. These distinct kinetic patterns may reflect fundamental biological differences in the mechanisms of action of cytotoxic chemotherapy and immune checkpoint inhibition. The marked early reduction in ctDNA observed with chemotherapy may, at least in part, reflect the rapid induction of tumor-cell death and consequent reduction in the release of tumor-derived DNA into the circulation. Conversely, the more modest early ctDNA decline observed with pembrolizumab may be related to the delayed and heterogeneous nature of immune-mediated tumor control, in which clinical benefit depends on the activation and expansion of antitumor T-cell responses rather than on direct cytotoxicity [5,6,10,16].

The relationship between ctDNA kinetics and treatment efficacy may also be influenced by the underlying biological characteristics of the tumor. In particular, intrinsic or acquired chemoresistance could result in limited durable benefit despite an early reduction in ctDNA, because chemotherapy may induce substantial initial tumor-cell killing without effectively eliminating resistant subclones. In contrast, immune checkpoint inhibition may exert a more selective pressure on immunologically susceptible tumor clones, potentially resulting in slower but more durable molecular responses in patients with an effective antitumor immune response. Consistent with this hypothesis, early ctDNA increases during immune checkpoint inhibition have been associated with shorter PFS and OS in advanced urothelial carcinoma, whereas ctDNA decreases or clearance are associated with durable clinical benefit.

The potential interaction between ctDNA and the PD-1/PD-L1 axis is particularly relevant. PD-L1 expression represents an established, although imperfect, biomarker of sensitivity to immune checkpoint inhibition. Nevertheless, available evidence does not support interpreting ctDNA levels simply as a surrogate for PD-L1 expression. Notably, in KEYNOTE-361, the associations between baseline ctDNA or early ctDNA changes and clinical outcomes in the pembrolizumab arm remained robust after adjustment for PD-L1 and tumor mutational burden, suggesting that ctDNA may capture biological information that is at least partly complementary to these conventional biomarkers.

Thus, ctDNA kinetics during immunotherapy may represent a composite marker of tumor burden and treatment-induced evolutionary dynamics rather than a simple measure of tumor shrinkage. This interpretation may also explain why ctDNA changes showed stronger associations with long-term outcomes under pembrolizumab despite larger absolute ctDNA reductions with chemotherapy. Importantly, however, these mechanistic hypotheses remain exploratory and require prospective validation through integrated analyses combining serial ctDNA, tumor genomics, PD-L1 expression, immune profiling, and radiographic response.

Collectively, these findings highlight distinct patterns of ctDNA kinetics between immunotherapy and chemotherapy, while also underscoring current limitations in relying on ctDNA alone for clinical decision-making in metastatic urothelial cancer [16].

From a diagnostic and clinical utility perspective, systematic evidence suggests that ctDNA provides moderate accuracy for predicting recurrence and metastatic progression. In a separate pooled analysis of nine studies, sensitivity was 0.68, specificity was 0.76, and the area under the curve was 0.75, indicating meaningful but still incomplete discriminative performance. Although ctDNA reflects real-time tumor dynamics and may enhance risk stratification, its standalone predictive value remains insufficient to replace conventional imaging and clinical assessment. Consequently, current evidence supports its use as an adjunctive tool rather than a fully independent decision-making biomarker [20]. A comprehensive summary of the clinical applications and prognostic implications of ctDNA according to disease stage is reported in Table 3.

## 3. ctDNA-Guided Treatment Strategies in Muscle-Invasive Bladder Cancer

The clinical integration of circulating tumor DNA (ctDNA) into the management of muscle-invasive bladder cancer (MIBC) is progressively shifting the paradigm from static, stage-based decision-making toward dynamic, molecularly informed treatment strategies. Beyond its prognostic relevance, ctDNA is increasingly being explored as an actionable biomarker for risk-adapted perioperative management, enabling both treatment escalation in patients with molecular residual disease (MRD) and potential de-escalation in those with sustained ctDNA negativity. In the adjuvant setting, postoperative ctDNA positivity consistently identifies a subgroup of patients at substantially higher risk of recurrence, often preceding radiological relapse by several months and thereby defining a clinically meaningful window for early intervention [12].

Collectively, these data support a transition toward longitudinal molecular surveillance as the backbone of postoperative treatment allocation in MIBC.

In earlier disease stages and metastatic settings, ctDNA is emerging as a complementary tool for real-time assessment of treatment response and therapeutic sensitivity, although its role in guiding routine clinical decisions remains investigational. In the neoadjuvant context, findings indicate that ctDNA dynamics closely mirror pathological response to immune checkpoint inhibition, with ctDNA clearance associated with durable disease control and persistently negative ctDNA identifying patients with particularly favorable outcomes who may ultimately benefit from treatment de-escalation or organ-preserving strategies [15].

In metastatic urothelial carcinoma, exploratory analyses suggest that ctDNA levels are associated with clinical outcomes. However, the lack of independent prognostic value beyond radiographic response in multivariable models highlights current limitations in clinical applicability [16].

## 4. Barriers to Clinical Implementation of ctDNA

Despite its strong prognostic and emerging predictive relevance in muscle-invasive and metastatic urothelial carcinoma, the clinical translation of circulating tumor DNA (ctDNA) remains constrained by several biological, technical, and methodological barriers. In particular, the lack of assay harmonization across platforms—including tumor-informed versus tumor-naïve approaches, differences in sequencing depth, and gene panel design—introduces substantial variability in detection performance, limiting inter-study comparability and hindering routine clinical adoption [21]. Moreover, the absence of standardized definitions for ctDNA positivity and clinically validated thresholds prevents its consistent integration into treatment algorithms, thereby restricting its role in prospective decision-making frameworks.

Biological factors further complicate clinical translation, as ctDNA release is highly heterogeneous and influenced by tumor burden, metastatic distribution, vascularity, and treatment exposure. This results in variable detectability across disease stages and increases the risk of false-negative findings, particularly in low-volume or minimal residual disease settings [22]. In addition, ctDNA kinetics remain incompletely characterized, and optimal sampling schedules in perioperative, surveillance, and metastatic contexts are not yet standardized, limiting its reproducibility as a longitudinal monitoring tool within clinical workflows.

From a clinical integration perspective, although ctDNA consistently correlates with recurrence risk and treatment response, its incremental value beyond established clinical, pathological, and radiological parameters remains inconsistent when incorporated into multivariable models [23]. In metastatic urothelial carcinoma, its independent contribution appears particularly limited once imaging-based response is accounted for, reinforcing its context-dependent performance across disease stages. Importantly, most available evidence derives from exploratory analyses or post hoc evaluations rather than prospective interventional studies designed to demonstrate outcome improvement through ctDNA-guided decision-making.

Finally, key translational gaps persist, including the lack of validated intervention thresholds, uncertainty regarding longitudinal monitoring strategies, and limited health-economic data supporting widespread implementation in routine clinical practice [24]. Consequently, while ctDNA represents a powerful biomarker for disease monitoring and risk stratification, its current role should be viewed as complementary to, rather than a replacement for, established clinical, pathological, and imaging-based decision-making tools within a multimodal precision oncology framework.

## 5. Emerging Liquid Biopsy Strategies in Bladder Cancer

Emerging evidence in urothelial bladder cancer (UBC) highlights the critical role of translational and clinical liquid biopsy strategies across disease stages. Specifically, tumor-informed circulating tumor DNA (ctDNA) and multi-compartment assays are refining risk stratification, guiding response-adapted bladder-sparing approaches, and optimizing perioperative decision-making through enhanced minimal residual disease (MRD) detection. Simultaneously, integrating tumor genomic profiling with serial ctDNA monitoring is emerging as a strategy to refine biological risk stratification, potentially identifying candidates for treatment de-escalation or intensified surveillance [25].

In the HCRN GU 20-444 trial, patients achieving a clinically defined complete response (cCR) after TURBT and pembrolizumab monotherapy were able to safely omit radical cystectomy while maintaining favorable outcomes. Furthermore, early ctDNA clearance post-treatment strongly predicted improved metastasis-free and overall survival, validating ctDNA as a robust early biomarker for durable disease control [26]. Complementing these findings, integrated analyses from the RETAIN trials confirmed that post-treatment ctDNA negativity correlates with a significantly reduced risk of distant metastasis. However, it proved less sensitive for detecting isolated intravesical recurrence, highlighting current limitations in capturing purely local residual disease [27].

Beyond binary MRD assessments, studies are now exploring the value of quantitative ctDNA thresholds prior to radical cystectomy. Preoperative ctDNA levels correlate with persistent post-surgical positivity, effectively stratifying patients into distinct biological risk groups. This suggests that continuous, quantitative ctDNA evaluation could better inform surgical decision-making and perioperative treatment planning [28].

Another major investigative focus is the integration of plasma ctDNA with urinary tumor DNA (utDNA) in multi-compartment liquid biopsy strategies. Translational data from the NIAGARA trial indicate that perioperative utDNA dynamics are strongly associated with event-free survival and pathological response. Combining ctDNA and utDNA assessments improves the stratification of residual disease and tumor stage at cystectomy, underscoring the complementary value of plasma and urine in capturing heterogeneity [29]. This multi-modal approach is also central to the CONSOLIDATE phase I/II study, which evaluates enfortumab vedotin combined with radiotherapy in locally advanced muscle-invasive disease. By incorporating paired ctDNA and utDNA analyses, the trial aims to characterize MRD dynamics and correlate molecular clearance with clinical outcomes [30].

In earlier disease stages, particularly carcinoma in situ (CIS), prospective data demonstrate the feasibility of blood- and urine-based ctDNA profiling for non-invasive monitoring. Notably, urine demonstrates higher sensitivity than plasma in this setting, with longitudinal changes correlating with molecular progression and clinical outcomes. This establishes urine-derived ctDNA as a highly informative tool for non–muscle-invasive disease, where conventional surveillance is both invasive and biologically limited [31]. The relationship between ctDNA and high-risk histological or clinical subgroups also deserves consideration. CIS represents a particularly challenging setting for plasma ctDNA detection because it may be associated with limited tumor volume and predominantly mucosal disease, potentially resulting in lower systemic DNA shedding. Accordingly, a negative plasma ctDNA result in patients with CIS should not be interpreted as evidence of the absence of residual disease. In this context, urinary tumor DNA may provide greater sensitivity because it is anatomically closer to the tumor compartment [4,31,32]. Similarly, urothelial carcinomas may display divergent or mixed histological differentiation, including squamous or glandular differentiation and other variant histologies. These tumors may differ in genomic landscape, tumor-cell turnover and biological behavior, potentially affecting ctDNA detectability and the relationship between ctDNA levels and clinical outcomes. However, evidence specifically addressing ctDNA performance according to histological subtype or mixed histology in MIBC remains limited. Future prospective studies incorporating histological subtype, genomic characteristics, and longitudinal ctDNA measurements will be important to determine whether specific variant histologies exhibit distinct patterns of ctDNA release or clearance.

Advancements in ultra-sensitive MRD detection are further highlighted by the MONSTAR-SCREEN-3 project. This study demonstrates the feasibility and broad genomic coverage of tumor-informed, whole-genome sequencing–based ctDNA assays. These ultra-sensitive platforms can detect low-level ctDNA, track postoperative clearance dynamics, and potentially anticipate radiographic recurrence, emphasizing the growing importance of personalized assay designs [33].

Finally, real-world evidence (RWE) from large electronic health record (EHR) cohorts confirms the strong prognostic value of post-operative ctDNA in routine clinical practice. Post-surgical ctDNA positivity is consistently associated with markedly inferior overall and disease-free survival, providing prognostic resolution beyond standard pathologic complete response (pCR). Notably, ctDNA identifies a high-risk subgroup of non-pCR patients who experience particularly poor outcomes despite curative-intent surgery. This reinforces the complementary role of liquid biopsies alongside histopathology, while emphasizing the need for prospective validation before widespread clinical implementation [34].

## 6. Future Perspectives: Artificial Intelligence and Multi-Omics Integration

Looking ahead, the clinical role of ctDNA is expected to evolve from a biomarker of molecular relapse toward a dynamic platform for treatment adaptation. Recent ctDNA-guided trials have introduced the concept of molecularly driven escalation and de-escalation strategies, in which therapeutic intensity may be adapted according to real-time assessment of residual disease rather than conventional clinicopathological risk factors alone [11,12,13].

**Figure 2:**
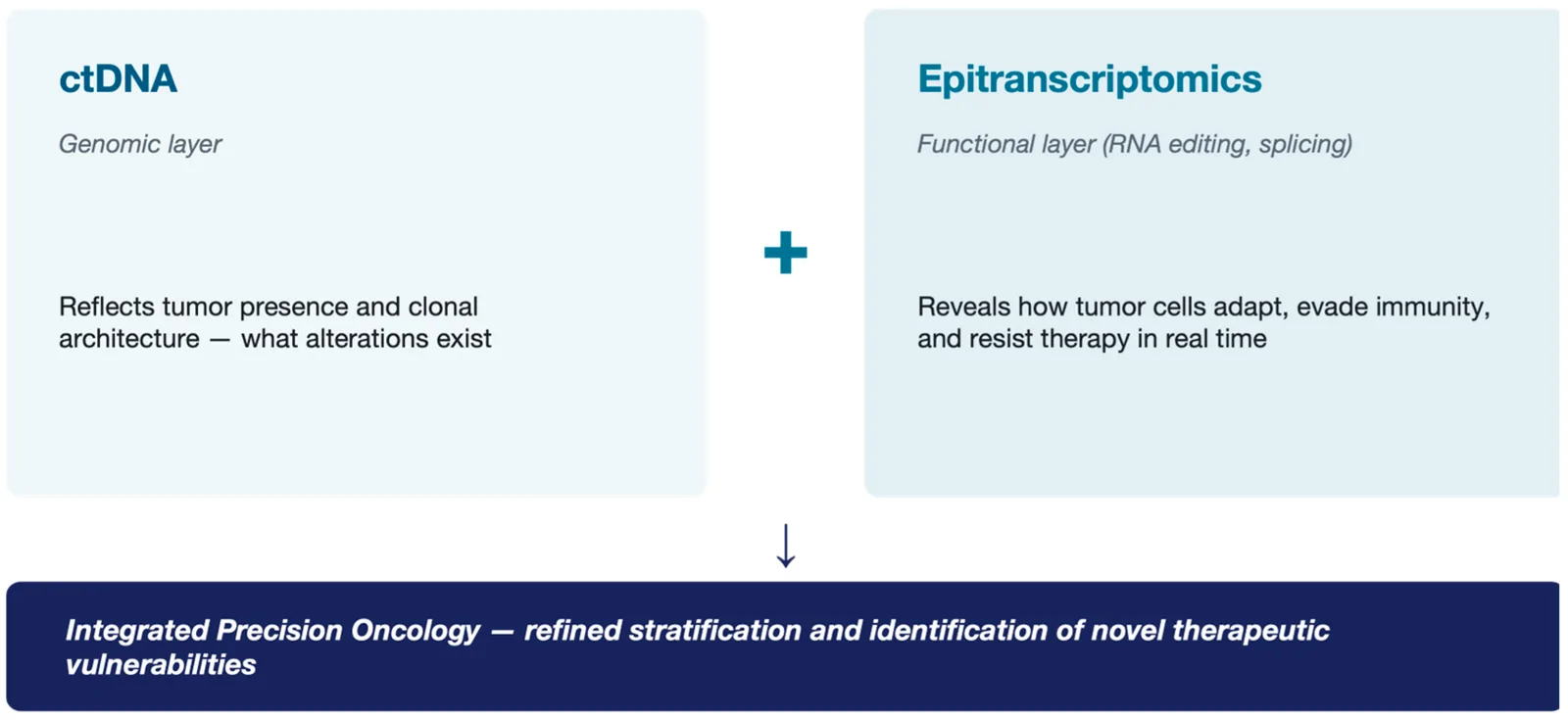
Integration of ctDNA genomics and epitranscriptomics for precision oncology in muscle-invasive bladder cancer. Abbreviations: ctDNA, circulating tumor DNA; RNA, ribonucleic acid.

Future developments will likely rely on the integration of multiple biological compartments and molecular layers. The combination of plasma ctDNA with urinary tumor DNA (utDNA) may improve the characterization of both systemic and local disease persistence, particularly in bladder-preserving strategies and early relapse monitoring. Furthermore, integrating genomic information derived from ctDNA with functional molecular layers, including epitranscriptomic and proteomic profiles, may provide additional insights into tumor evolution, immune escape, and mechanisms of therapeutic resistance [4,24,29,30,31,32,35] (Figure 2).

Artificial intelligence (AI) and machine learning approaches may represent a key enabling technology for the clinical translation of these complex datasets. AI-driven algorithms integrating longitudinal ctDNA kinetics, radiographic imaging, pathological features, and multi-omic information could improve individualized prediction of recurrence risk and treatment response beyond any single biomarker approach. Prospective trials incorporating these integrative frameworks will be required to validate their clinical utility, establish reproducible intervention thresholds, and define their role within routine precision oncology workflows.

## 7. Conclusions

Circulating tumor DNA analysis has rapidly evolved from a purely prognostic exploratory tool into a robust, dynamic biomarker capable of guiding perioperative and adjuvant treatment decisions in muscle-invasive bladder cancer (MIBC). Prospective data from practice-changing trials firmly establish the clinical utility of ctDNA-directed immunotherapy escalation in patients with molecular residual disease, heralding a paradigm shift from static, stage-based management to true precision oncology. Nevertheless, full clinical integration requires overcoming current limitations, including assay harmonization, cost-effectiveness analyses, and the standardization of intervention thresholds. Furthermore, emerging multi-compartment approaches—such as integrating plasma ctDNA with urinary tumor DNA (utDNA)—alongside novel functional layers like epitranscriptomics, promise to further refine patient stratification. Ultimately, longitudinal liquid biopsies will likely serve as the backbone of future bladder cancer management, perfectly complementing conventional clinical, pathological, and radiological workflows.

## Figures and Tables

**Table 1:** Overview of key prospective and exploratory clinical studies evaluating circulating tumor DNA (ctDNA) across the muscle-invasive and metastatic bladder cancer continuum. Abbreviations: AUC, area under the curve; ctDNA, circulating tumor DNA; DFS, disease-free survival; HR, hazard ratio; MIBC, muscle-invasive bladder cancer; MRD, minimal residual disease; OS, overall survival.

Study	Disease Setting	ctDNA Platform	Timing of Assessment	Main Findings
CheckMate 274	MIBC (post-cystectomy adjuvant)	Tumor-informed ctDNA (exploratory)	Postoperative/longitudinal	ctDNA positivity (~40.6%) associated with worse DFS; benefit from adjuvant nivolumab higher in ctDNA+ patients (HR 0.35)
IMvigor011	MIBC (adjuvant ctDNA-guided therapy)	Tumor-informed serial ctDNA	Post-surgery surveillance	ctDNA-guided atezolizumab improved DFS (HR 0.64) and OS (HR 0.59); ctDNA-negative patients had excellent outcomes without therapy
TOMBOLA	MIBC (postoperative MRD monitoring)	Tumor-informed ctDNA	Longitudinal post-surgery	58% ctDNA-positive within 2 years; molecular relapse preceded imaging by ~90 days
ABACUS	MIBC (neoadjuvant immunotherapy)	ctDNA sequencing-based assay	Baseline, post-therapy, post-surgery	ctDNA decreased from 63% to 14% after surgery; ctDNA clearance associated with no relapse
KEYNOTE-361	Metastatic urothelial carcinoma	Plasma ctDNA profiling	Baseline + early on-treatment	ctDNA associated with response and survival under pembrolizumab but not chemotherapy; no independent prognostic value

**Table 2:** Summary of ctDNA assessment methodologies, cutoff definitions, and clinical significance across key clinical studies in urothelial cancer. Abbreviations: ctDNA, circulating tumor DNA; MTM/mL, mean tumor molecules per milliliter; OS, overall survival; PFS, progression-free survival; RFS, relapse-free survival.

Study	ctDNA Assessment/Cutoff Definition	Clinical Significance
CheckMate 274	Tumor-informed ctDNA; positivity based on detection of tumor-specific variants rather than a universal MTM/mL threshold	ctDNA positivity after surgery identified patients at higher risk of recurrence and greater relative benefit from adjuvant nivolumab [11]
IMvigor011	Serial tumor-informed ctDNA assessment; patients were classified according to detectable versus undetectable ctDNA during postoperative surveillance	ctDNA positivity identified patients eligible for randomization to adjuvant atezolizumab or placebo; persistent ctDNA negativity was associated with excellent outcomes without treatment [13]
TOMBOLA	Serial tumor-informed ctDNA; longitudinal detection/clearance rather than a single universal quantitative cutoff	Persistent or newly detectable ctDNA identified patients at increased risk of recurrence, whereas ctDNA negativity was associated with favorable RFS [12]
ABACUS	Tumor-informed ctDNA assessment at baseline, after neoadjuvant treatment, and after surgery; positivity based on assay detection	Progressive ctDNA clearance was associated with pathological response and favorable long-term outcomes [15]
KEYNOTE-361	Plasma ctDNA quantified longitudinally; baseline levels and early changes were analyzed as continuous/dynamic measures rather than using a single universal clinical cutoff	Baseline ctDNA and early ctDNA changes were associated with response, PFS, and OS in the pembrolizumab arm [16]

**Table 3:** Overview of clinical applications, prognostic implications, and evidence levels of circulating tumor DNA (ctDNA) across the bladder cancer disease continuum. Abbreviations: AUC, area under the curve; CIS, carcinoma in situ; ctDNA, circulating tumor DNA; DFS, disease-free survival; MIBC, muscle-invasive bladder cancer; MRD, minimal residual disease; NMIBC, non–muscle-invasive bladder cancer; OS, overall survival.

Disease Stage	ctDNA Status/Pattern	Associated Outcomes	Strength of Evidence	Clinical Interpretation
Post-cystectomy (MIBC)	ctDNA positive vs. negative	ctDNA positivity strongly associated with worse DFS and OS; ctDNA often precedes radiological recurrence by months	Strong (multiple trials + meta-analyses)	Robust marker of minimal residual disease (MRD); identifies high-risk patients for relapse
Post-cystectomy (adjuvant setting)	ctDNA-guided treatment vs. standard approach	Improved DFS and OS in ctDNA-positive patients receiving immunotherapy (e.g., atezolizumab, nivolumab)	High (phase III + exploratory analyses)	Predictive role emerging for selection of adjuvant immunotherapy
Postoperative surveillance (longitudinal MRD monitoring)	Rising or persistent ctDNA vs. clearance	Molecular recurrence precedes imaging (~weeks–months); ctDNA clearance associated with excellent outcomes	Strong (prospective + observational studies)	Enables early detection of relapse and dynamic risk stratification
Neoadjuvant setting (MIBC)	ctDNA clearance vs. persistence after therapy	ctDNA clearance correlates with pathological response and durable disease control; persistence associated with relapse	Moderate–strong	Real-time indicator of treatment response and residual disease burden
Metastatic urothelial carcinoma	Baseline ctDNA and early dynamics	Associated with response and survival under immunotherapy; limited independent prognostic value when adjusted for imaging	Moderate	Useful for monitoring but not yet a standalone decision tool
Non–muscle-invasive bladder cancer (NMIBC)	Urine ctDNA positivity vs. negativity	Higher sensitivity in urine than plasma; associated with disease progression in CIS and early lesions	Emerging	Promising non-invasive surveillance tool, still investigational
Post-treatment kinetics (all stages)	ctDNA clearance vs. increase	Clearance associated with improved survival; increasing ctDNA predicts progression	Strong	Dynamic (longitudinal) changes more informative than single timepoint
Overall diagnostic performance (mixed cohorts)	ctDNA detection for recurrence/metastasis	Sensitivity 0.68, specificity 0.76, AUC 0.75	Moderate	Adjunctive biomarker; not sufficient to replace imaging or cystoscopy

## Data Availability

No new data were created or analyzed in this study. Data sharing is not applicable to this article.

## Abbreviations

The following abbreviations are used in this manuscript:

AI	Artificial intelligence
ctDNA	Circulating tumour DNA
MIBC	Muscle-invasive-bladder-cancer
BC	Bladder cancer
NGS	Next-Generation Sequencing
TMB	Tumor Mutational Burden
OS	Overall Survival
DFS	Disease-free Survival
RFS	Recurrence-free Survival

## References

[1] Dyrskjøt L., Hansel D.E., Efstathiou J.A., Knowles M., Galsky M., Teoh J., Theodorescu D. (2023). Bladder cancer. Nat. Rev. Dis. Primers.

[2] Holzbeierlein J., Bixler B.R., Buckley D.I., Chang S.S., Holmes R.S., James A.C., Kirkby E., McKiernan J.M., Schuckman A. (2024). Treatment of Non-Metastatic Muscle-Invasive Bladder Cancer: AUA/ASCO/SUO Guideline (2017; Amended 2020, 2024). J. Urol..

[3] Stein J.P., Lieskovsky G., Cote R., Groshen S., Feng A.-C., Boyd S., Skinner E., Bochner B., Thangathurai D., Mikhail M. (2001). Radical Cystectomy in the Treatment of Invasive Bladder Cancer: Long-Term Results in 1054 Patients. J. Clin. Oncol..

[4] Rose K.M., Huelster H.L., Meeks J.J., Faltas B.M., Sonpavde G.P., Lerner S.P., Ross J.S., Spiess P.E., Grass G.D., Jain R.K. (2023). Circulating and urinary tumour DNA in urothelial carcinoma—Upper tract, lower tract and metastatic disease. Nat. Rev. Urol..

[5] Wan J.C.M., Massie C., Garcia-Corbacho J., Mouliere F., Brenton J.D., Caldas C., Pacey S., Baird R., Rosenfeld N. (2017). Liquid biopsies come of age: Towards implementation of circulating tumour DNA. Nat. Rev. Cancer.

[6] Lindskrog S.V., Strandgaard T., Nordentoft I., Galsky M.D., Powles T., Agerbæk M., Jensen J.B., Alix-Panabières C., Dyrskjøt L. (2025). Circulating tumour DNA and circulating tumour cells in bladder cancer—From discovery to clinical implementation. Nat. Rev. Urol..

[7] Christensen E., Birkenkamp-Demtröder K., Sethi H., Shchegrova S., Salari R., Nordentoft I., Wu H.-T., Knudsen M., Lamy P., Lindskrog S.V. (2019). Early Detection of Metastatic Relapse and Monitoring of Therapeutic Efficacy by Ultra-Deep Sequencing of Plasma Cell-Free DNA in Patients With Urothelial Bladder Carcinoma. J. Clin. Oncol..

[8] Powles T., Assaf Z.J., Davarpanah N., Banchereau R., Szabados B.E., Yuen K.C., Grivas P., Hussain M., Oudard S., Gschwend J.E. (2021). ctDNA guiding adjuvant immunotherapy in urothelial carcinoma. Nature.

[9] Maia M.C., Salgia M., Pal S.K. (2020). Harnessing cell-free DNA: Plasma circulating tumour DNA for liquid biopsy in genitourinary cancers. Nat. Rev. Urol..

[10] Birkenkamp-Demtröder K., Christensen E., Nordentoft I., Knudsen M., Taber A., Høyer S., Lamy P., Agerbæk M., Jensen J.B., Dyrskjøt L. (2018). Monitoring Treatment Response and Metastatic Relapse in Advanced Bladder Cancer by Liquid Biopsy Analysis. Eur. Urol..

[11] Galsky M.D., Gschwend J.E., Milowsky M.I., Schenker M., Valderrama B.P., Tomita Y., Bamias A., Lebret T., Shariat S.F., Park S.H. (2026). Adjuvant nivolumab versus placebo for high-risk muscle-invasive urothelial carcinoma: 5-year efficacy and ctDNA results from CheckMate 274. Ann. Oncol..

[12] Dyrskjøt L., Birkenkamp-Demtröder K., Nordentoft I., Strandgaard T., Lindskrog S.V., Milling R.V., Körner S.K., Brandt S.B., Knudsen M., Andreasen T.G. (2026). ctDNA-guided immunotherapy following radical cystectomy for muscle-invasive bladder cancer: Results from the TOMBOLA trial. Ann. Oncol..

[13] Powles T., Kann A.G., Castellano D., Gross-Goupil M., Nishiyama H., Bracarda S., Bjerggaard Jensen J., Makaroff L., Jiang S., Ku J.H. (2025). ctDNA-Guided Adjuvant Atezolizumab in Muscle-Invasive Bladder Cancer. N. Engl. J. Med..

[14] Powles T., Grindheim J., Yilmaz M., Rallis G., Gumus M., Buttigliero C., Bonfill T., Nole F., Seo H.K., Matouskova M. (2026). Circulating tumor (ct)DNA-guided adjuvant atezolizumab (atezo) in muscle-invasive bladder cancer (MIBC): Exploratory analysis of ctDNA dynamics in the IMvigor011 trial. J. Clin. Oncol..

[15] Szabados B., Kockx M., Assaf Z.J., Van Dam P.-J., Rodriguez-Vida A., Duran I., Crabb S.J., Van Der Heijden M.S., Pous A.F., Gravis G. (2022). Final Results of Neoadjuvant Atezolizumab in Cisplatin-ineligible Patients with Muscle-invasive Urothelial Cancer of the Bladder. Eur. Urol..

[16] Powles T., Chang Y.-H., Yamamoto Y., Munoz J., Reyes-Cosmelli F., Peer A., Cohen G., Yu E.Y., Lorch A., Bavle A. (2024). Pembrolizumab for advanced urothelial carcinoma: Exploratory ctDNA biomarker analyses of the KEYNOTE-361 phase 3 trial. Nat. Med..

[17] Ma Q., Hou S., Ma H., Gao J., Song D. (2025). Prognostic significance of circulating tumor DNA in urothelial carcinoma patients undergoing immune checkpoint inhibitor therapy: A systematic review and meta-analysis. Front. Immunol..

[18] Liu H., Chen J., Huang Y., Zhang Y., Ni Y., Xu N., Zhao F., Tang Y., Liu H., Sun G. (2024). Prognostic significance of circulating tumor DNA in urothelial carcinoma: A systematic review and meta-analysis. Int. J. Surg..

[19] Pon Avudaiappan A., Ganiyani M.A., Agarwal A., Valagni G., Pustake M., Khosla A.A., Bangia V., Jatwani K., Sonpavde G.P., Manoharan M. (2026). Prognostic value of circulating tumor DNA in muscle-invasive bladder cancer treated with radical cystectomy: A systematic review and meta-analysis. J. Clin. Oncol..

[20] Lu Y., Wu H., Jiang J., Bao S., Ling C. (2025). The Value of Circulating Tumor DNA in the Prognostic Diagnosis of Bladder Cancer: A Systematic Review and Meta-Analysis. Urol. Int..

[21] Sultana T., Zamborlini A., Cristofari G., Lesage P. (2017). Integration site selection by retroviruses and transposable elements in eukaryotes. Nat. Rev. Genet..

[22] Bettegowda C., Sausen M., Leary R.J., Kinde I., Wang Y., Agrawal N., Bartlett B.R., Wang H., Luber B., Alani R.M. (2014). A. Detection of Circulating Tumor DNA in Early- and Late-Stage Human Malignancies. Sci. Transl. Med..

[23] Peng Y., Mei W., Ma K., Zeng C. (2021). Circulating Tumor DNA and Minimal Residual Disease (MRD) in Solid Tumors: Current Horizons and Future Perspectives. Front. Oncol..

[24] Eisenberg E., Levanon E.Y. (2018). A-to-I RNA editing—Immune protector and transcriptome diversifier. Nat. Rev. Genet..

[25] Aydogdu C., Wang B., McSweeney S.T., Diaz G., Baer M., Guzman T., Ram Dewala S., Arya R., Davis L.E., Nizam A. (2026). Integrating genomic profiling and circulating tumor DNA monitoring to optimize surveillance strategies in muscle-invasive bladder cancer. J. Clin. Oncol..

[26] Anker J.F., King J., Tripathi A., Izadmehr S., Hashemi-Sadraei N., Pal S.K., Salous T., Zhao Q., Yu M., Afterman D. (2026). Phase 2 trial of pembrolizumab (P) with response-guided bladder-sparing in patients with muscle-invasive bladder cancer (MIBC; HCRN GU 20-444). J. Clin. Oncol..

[27] Ghatalia P., Ross E.A., Zibelman M.R., Anari F., Abbosh P., Herberts C., Mille P.J., Rose T.L., Cole S., Mark J.R. (2026). Circulating tumor DNA (ctDNA) to guide response-adapted bladder preservation in muscle invasive bladder cancer (MIBC): Integrated analysis of the RETAIN trials. J. Clin. Oncol..

[28] Ayasun R., Miller E.J., Weintraub L.S., Wu L., Argulian A., Elikan A., Otten T.J., Heath E., Ganta T., Atiq S.O. (2026). Quantitative pre-cystectomy ctDNA levels for identification of ctDNA-positive patients with surgically curable loco-regional disease versus those with occult micrometastatic progression. J. Clin. Oncol..

[29] Van Der Heijden M.S., Galsky M.D., Joshi R., Catto J.W.F., Meeks J.J., Nishiyama H., Al-Ahmadie H., Antonuzzo L., Quang T.V.U., Zakharia Y. (2026). Urinary tumor DNA (utDNA) and circulating tumor DNA (ctDNA) in patients (pts) with muscle-invasive bladder cancer (MIBC) who received perioperative durvalumab (D) in NIAGARA. J. Clin. Oncol..

[30] Carriere P.P.J., Alhalabi O., Kamat A.M., Gao J., Goswami S., Campbell M.T., Shah A.Y., Jiang C.Y., Glover M., Bree K. (2026). CONSOLIDATE: A phase I/II study of radiotherapy combined with enfortumab vedotin (EV) for locally advanced bladder cancer with paired translational ctDNA and utDNA. J. Clin. Oncol..

[31] Zhang R., Tang H., Jin D., Qian L., Xie F., Zhuang G., Jia S., Chen H. (2026). Blood- and urine-based ctDNA profiling and monitoring in bladder carcinoma in situ. J. Clin. Oncol..

[32] Nuzzo P.V., Berchuck J.E., Korthauer K., Spisak S., Nassar A.H., Abou Alaiwi S., Chakravarthy A., Shen S.Y., Bakouny Z., Boccardo F. (2020). Detection of renal cell carcinoma using plasma and urine cell-free DNA methylomes. Nat. Med..

[33] Tsukahara S., Yajima S., Shiota M., Osawa T., Kojima T., Kato T., Tanaka N., Hayashi Y., Masuda H., Abe T. (2026). Ultra-sensitive molecular residual disease detection via tumor-informed whole-genome sequencing-based ctDNA assay in resectable urothelial cancer in the MONSTAR-SCREEN-3 project. J. Clin. Oncol..

[34] Patel K., Zhao D., Fidyk E., Hankinson E.A., Mehta A.M., Pesavento M., Sujenthiran A. (2026). Prognostic value of post-operative circulating tumor DNA (ctDNA) in real-world treatment and outcomes among patients (pts) with muscle invasive (MIBC) and locally advanced (LA) bladder cancer (BC). J. Clin. Oncol..

[35] Han L., Diao L., Yu S., Xu X., Li J., Zhang R., Yang Y., Werner H.M.J., Eterovic A.K., Yuan Y. (2015). The Genomic Landscape and Clinical Relevance of A-to-I RNA Editing in Human Cancers. Cancer Cell.

